# Age at first childbirth and newly diagnosed diabetes among postmenopausal women: a cross-sectional analysis of the Brazilian Longitudinal Study of Adult Health (ELSA-Brasil): Idade no primeiro parto e diabetes recentemente diagnosticada em mulheres pós-menopáusicas: uma análise transversal do Estudo Longitudinal de Saúde do Adulto (ELSA-Brasil)

**DOI:** 10.1590/1516-3180.2017.0015240217

**Published:** 2017-04-03

**Authors:** James Yarmolinsky, Bruce Bartholow Duncan, Sandhi Maria Barreto, Maria de Fátima Sander Diniz, Dora Chor, Maria Inês Schmidt

**Affiliations:** I MSc. Research Assistant, Postgraduate Program on Epidemiology, School of Medicine, Universidade Federal do Rio Grande do Sul (UFRGS), Porto Alegre (RS), Brazil.; II MD, PhD. Professor, Department of Social Medicine and Postgraduate Program on Epidemiology, School of Medicine, Universidade Federal do Rio Grande do Sul (UFRGS), and Hospital de Clínicas de Porto Alegre (HCPA), Porto Alegre (RS), Brazil.; III MD, PhD. Professor, Department of Social and Preventive Medicine and Postgraduate Program in Public Health, Universidade Federal de Minas Gerais (UFMG), Belo Horizonte (MG), Brazil.; IV MD, PhD. Professor, Department of Internal Medicine, School of Medicine, Universidade Federal de Minas Gerais (UFMG), Belo Horizonte (MG), Brazil.; V MD, PhD. Professor, Escola Nacional de Saúde Pública (ENSP), Fundação Oswaldo Cruz (Fiocruz), Rio de Janeiro (RJ), Brazil.

**Keywords:** Diabetes mellitus, type 2, Pregnancy in adolescence, Reproductive behavior, Postmenopause, Reproducibility of results

## Abstract

**CONTEXT AND OBJECTIVE:**

It has been reported that earlier age at first childbirth may increase the risk of adult-onset diabetes among postmenopausal women, a novel finding with important public health implications. To date, however, no known studies have attempted to replicate this finding. We aimed to test the hypothesis that age at first childbirth is associated with the risk of adult-onset diabetes among postmenopausal women.

**DESIGN AND SETTING:**

Cross-sectional analysis using baseline data from 2919 middle-aged and elderly postmenopausal women in the Brazilian Longitudinal Study of Adult Health (ELSA-Brasil).

**METHODS:**

Age at first childbirth was determined from self-reporting and newly diagnosed diabetes through a 2-hour 75-g oral glucose tolerance test and/or glycated hemoglobin. Logistic regression was performed to examine associations between age at first childbirth and newly diagnosed diabetes among postmenopausal women.

**RESULTS:**

We did not find any association between age at first childbirth and diabetes, either when minimally adjusted for age, race and study center (odds ratio, OR [95% confidence interval, CI]: ≤ 19 years: 1.15 [0.82-1.59], 20-24 years: 0.90 [0.66-1.23] and ≥ 30 years: 0.86 [0.63-1.17] versus 25-29 years; P = 0.36) or when fully adjusted for childhood and adult factors (OR [95% CI]: ≤ 19 years: 0.95 [0.67-1.34], 20-24 years: 0.78 [0.56-1.07] and ≥ 30 years: 0.84 [0.61-1.16] versus 25-29 years; P = 0.40).

**CONCLUSION:**

Our current analysis does not support the existence of an association between age at first childbirth and adult-onset diabetes among postmenopausal women, which had been reported previously.

## RESUMO

**CONTEXTO E OBJETIVO:**

Foi relatado que idade mais precoce no primeiro parto pode aumentar o risco do diabetes de início adulto entre mulheres na menopausa, um novo achado com implicações de saúde pública importantes. Até então, no entanto, nenhum estudo conhecido tentou replicar esta descoberta. Objetivou-se testar a hipótese de que a idade no primeiro parto está associada ao risco de diabetes de início na vida adulta em mulheres pós-menopáusicas.

**DESENHO DE ESTUDO E LOCAL:**

Análise transversal utilizando dados de base de 2.919 mulheres pós-menopáusicas de meia-idade e idosas no Estudo Longitudinal de Saúde do Adulto (ELSA-Brasil).

**MÉTODOS:**

A idade no primeiro parto foi determinada por autorrelato e diabetes recentemente diagnosticado por um teste de tolerância à glicose oral de 2 horas com 75 g e/ou hemoglobina glicada. A regressão logística foi realizada para examinar associações entre idade no primeiro parto e diabetes recentemente diagnosticada entre mulheres pós-menopáusicas.

**RESULTADOS:**

Não encontramos associação entre idade no primeiro parto e diabetes, quando ajustados minimamente para idade, raça e centro de estudo (*odds ratio*, OR [intervalo de confiança, IC 95%]: ≤ 19 anos: 1,15 [0,82-1,59], 20-24 anos: 0,90 [0,66-1,23], ≥ 30 anos: 0,86 [0,63-1,17] *versus* 25-29 anos, P = 0,36) ou quando totalmente ajustados para fatores infantis e adultos (OR [IC 95%]: ≤ 19 anos: 0,95 [0,67-1,34], 20-24 anos: 0,78 [0,56-1,07], ≥ 30 anos: 0,84 [0,61-1,16] *versus* 25-29 anos, P = 0,40).

**CONCLUSÃO:**

Nossa análise atual não apoia uma associação entre a idade no primeiro parto e o diabetes de início na vida adulta entre mulheres pós-menopáusicas, como relatado anteriormente.

## INTRODUCTION

It has been suggested that reproductive health factors over the course of life may play an important role in the risk of chronic disease in later life.^1^ In the context of type 2 diabetes, earlier age at menarche, higher parity and early menopause have all been linked to higher risk in later life.^2^^,^^3^^,^^4^^,^^5^ It has recently been reported by Kim et al. that postmenopausal Korean women who were ≤ 19 years of age at first childbirth, compared with women who were 25-29 years of age, presented increased odds of having type 2 diabetes (odds ratio, OR 1.492; 95% confidence interval, CI: 1.005-2.215) in analyses adjusted for a comprehensive panel of potential confounding factors.^6^ This novel finding, which would imply that the timing of first childbearing and the postmenopausal period would have a combined role in the etiology of diabetes, could have important public health implications and inform screening practices targeting women with adolescent pregnancies. To date, however, no known studies have attempted to replicate this finding.

## OBJECTIVE

Given the potential relevance of these findings, we thus aimed to examine the association of age at first childbirth with newly diagnosed adult-onset diabetes in a postmenopausal subset of a large Brazilian cohort of middle-aged and elderly individuals.

## METHODS

The Brazilian Longitudinal Study of Adult Health (in Portuguese, Estudo Longitudinal de Saude do Adulto, or ELSA-Brasil) is a prospective cohort study designed to investigate the distribution, determinants and consequences of diabetes and cardiovascular disease. The details of the study, including design, eligibility criteria, sources and recruitment methods, and the measurements obtained, have been described in detail elsewhere.^7^^,^^8^ The cohort comprises 15,105 civil servants, aged 35 to 74 years at baseline (2008-2010), who were sampled from universities or research institutions located in six cities in three different regions of Brazil. All data for the current cross-sectional analyses were collected at baseline. The study was approved by the local Research Ethics Committees of all the institutions involved. All participants provided written informed consent for their clinical records to be used in this study, prior to enrolment.

Briefly, postmenopausal status was defined as reporting not having experienced a menstrual cycle within the previous 12 months, and included natural or induced menopause. Age at first childbirth was determined from self-reporting. Newly diagnosed diabetes was defined as fasting blood glucose ≥ 7.0 mmol/l, 2-hour postload glucose ≥ 11.1 mmol/l, or HbA_1c_ (glycated hemoglobin) ≥ 6.5%. 

Among postmenopausal women with a history of childbirth, we excluded those with missing data (n = 646) and those reporting a previous diagnosis of diabetes or use of diabetes medication (n = 466). Consequently, our sample consisted of 2919 postmenopausal women.

We assessed the association of diabetes with age at first childbirth, with the latter categorized into four groups: ≤ 19, 20-24, 25-29 and ≥ 30 years, taking 25-29 as our reference category. In modeling, we first presented a model that was minimally adjusted for age, race and study center (Model 1), followed by models sequentially adjusted for childhood and adolescent factors that might confound any observed association (Model 2: maternal education and age at menarche), and then for adult socioeconomic and lifestyle factors that might confound or mediate any associations presented (Model 3: education level, family income, smoking, alcohol consumption, number of pregnancies and any use of hormone replacement therapy). Multivariable logistic regression was performed to generate ORs and 95% CIs for the association between age category at first childbirth and newly diagnosed adult-onset diabetes. All statistical tests were two-sided and significance was defined as P < 0.05. Statistical analyses were performed using SAS 9.4 (SAS Institute, Inc., Cary, North Carolina, USA).

## RESULTS

The participants in our sample were, on average, 57.3 years of age (standard deviation, SD: 6.7) and 9.9 years (SD: 7.1) postmenopausal. Generally, participants who reported earlier age at first childbirth were more likely to be black or mixed-race, have a lower education level and be a current smoker (not shown). At baseline, we found 352 cases (12.1%) of newly diagnosed diabetes.

In logistic regression analyses, we failed to find any association between age at first childbirth and newly diagnosed diabetes in either the model minimally adjusted for age, race and study center (OR [95% CI]: ≤ 19 years: 1.15 [0.82-1.59], 20-24 years: 0.90 [0.66-1.23] and ≥ 30 years: 0.86 [0.63-1.17] versus 25-29 years; P = 0.36) or in models sequentially adjusted for childhood and adolescent factors (OR [95% CI]: ≤ 19 years: 1.14 [0.82-1.59], 20-24 years: 0.90 [0.66-1.22] and ≥ 30 years: 0.86 [0.63-1.17] versus 25-29 years; P = 0.36) and adulthood socioeconomic and lifestyle factors (OR [95% CI]: ≤ 19 years: 0.95 [0.67-1.34], 20-24 years: 0.78 [0.56-1.07] and ≥ 30 years: 0.84 [0.61-1.16] versus 25-29 years, P = 0.40) (Table 1).

**Table 1:**
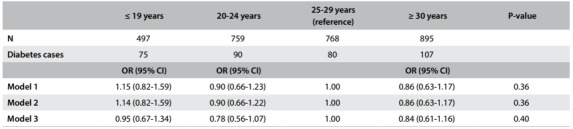
Association of age at first childbirth with newly-diagnosed diabetes in postmenopausal women, Brazilian Longitudinal Study of Adult Health (ELSA-Brasil), 2008-2010; n = 2919

Model 1: adjusted for age, race and study center; Model 2: + childhood and adolescent factors (maternal education and age at menarche); Model 3: + adulthood socioeconomic and lifestyle factors (education level, family income, smoking, alcohol consumption, number of pregnancies and any use of hormone replacement therapy). P-value represents the test for an overall association of age at first childbirth with newly-diagnosed diabetes. OR = odds ratio; CI = confidence interval.

Nor did we find any association between age at first childbirth and impaired fasting glucose (fasting glucose concentration ≥ 5.5 mmol/l and < 7.0 mmol/l), impaired glucose tolerance (two-hour postload glucose concentration ≥ 7.8 mmol/l and < 11.1 mmol/l) or previously diagnosed diabetes in analyses in all women independent of menopausal status, or in analyses that excluded those who did not experience a natural menopause (not shown).

## DISCUSSION

In this cross-sectional analysis on 2919 middle-aged and elderly postmenopausal women, we did not find any association between age at first childbirth and newly diagnosed adult-onset diabetes, which had been reported previously.

Some important strengths of our study, in relation to the previous analysis, deserve brief comment. Firstly, the extensive laboratory measurements used in ELSA-Brasil to ascertain previously unknown diabetes, including a centrally measured standard 75-g oral glucose tolerance test and measurements of glycated hemoglobin, enabled a broader and more sensitive assessment of adult-onset diabetes than in the previous analysis, which relied solely on fasting plasma glucose. Furthermore, exclusion of postmenopausal women with previously diagnosed diabetes from our analyses allowed us to more accurately test the hypothesis that earlier age at first childbirth was associated with development of diabetes within the postmenopausal period. This is in contrast to the analysis of Kim et al.,^6^ who included postmenopausal women with previously diagnosed diabetes in their analysis without knowledge of their duration of diabetes, which masked the age at onset of diabetes among the participants. This inability of their analysis to ascertain whether diabetes was diagnosed pre or postmenopausally makes their suggestion, i.e. that the postmenopausal metabolic milieu might explain their findings, questionable.

Thus, if the null finding in our study, in which diabetes was clearly diagnosed postmenopausally, were to be confirmed in further studies, it would suggest that the findings of Kim et al.^6^ could be attributable to residual or unmeasured confounding, ethnic differences in the etiology of diabetes, or chance.

## CONCLUSIONS

In conclusion, our analyses failed to replicate any association between age at first childbirth and diabetes among postmenopausal women, which had been reported previously. However, in the light of the increasingly recognized role of women’s reproductive health factors operating over the course of life, in shaping the risk of adult-onset diabetes, a putative biological role for adolescent pregnancy in the pathophysiology of diabetes should not be ruled out. Thus, further investigation of a potential link between age at first childbirth and type 2 diabetes in postmenopausal women, particularly in the context of a large-scale prospective study, remains warranted.
